# Blood Vitamin C Levels of Patients Receiving Immunotherapy and Relationship to Monocyte Subtype and Epigenetic Modification

**DOI:** 10.3390/epigenomes8020017

**Published:** 2024-04-30

**Authors:** Ben Topham, Millie de Vries, Maria Nonis, Rebecca van Berkel, Juliet M. Pullar, Nicholas J. Magon, Margreet C. M. Vissers, Margaret J. Currie, Bridget A. Robinson, David Gibbs, Abel Ang, Gabi U. Dachs

**Affiliations:** 1 Mackenzie Cancer Research Group, Department of Pathology and Biomedical Science, University of Otago Christchurch, Christchurch 8011, New Zealand; ben.topham@otago.ac.nz (B.T.); margaret.currie@otago.ac.nz (M.J.C.); bridget.robinson@cdhb.health.nz (B.A.R.); abel.ang@gmail.com (A.A.); 2 Mātai Hāora—Centre for Redox Biology and Medicine, Department of Pathology and Biomedical Science, University of Otago Christchurch, Christchurch 8011, New Zealand; juliet.pullar@otago.ac.nz (J.M.P.); margreet.vissers@otago.ac.nz (M.C.M.V.); 3 Canterbury Regional Cancer and Haematology Service, Te Whatu Ora Waitaha, Canterbury, Christchurch 8011, New Zealand; 4 Division of Cellular Medicine, School of Medicine, University of Dundee, Dundee DD1 4HN, UK

**Keywords:** metastatic melanoma, ascorbate, DNA methylation, monocytes

## Abstract

The treatment of metastatic melanoma has been revolutionised by immunotherapy, yet a significant number of patients do not respond, and many experience autoimmune adverse events. Associations have been reported between patient outcome and monocyte subsets, whereas vitamin C (ascorbate) has been shown to mediate changes in cancer-stimulated monocytes in vitro. We therefore investigated the relationship of ascorbate with monocyte subsets and epigenetic modifications in patients with metastatic melanoma receiving immunotherapy. Patients receiving immunotherapy were compared to other cancer cohorts and age-matched healthy controls. Ascorbate levels in plasma and peripheral blood-derived mononuclear cells (PBMCs), monocyte subtype and epigenetic markers were measured, and adverse events, tumour response and survival were recorded. A quarter of the immunotherapy cohort had hypovitaminosis C, with plasma and PBMC ascorbate levels significantly lower than those from other cancer patients or healthy controls. PBMCs from the immunotherapy cohort contained similar frequencies of non-classical and classical monocytes. DNA methylation markers and intracellular ascorbate concentration were correlated with monocyte subset frequency in healthy controls, but correlation was lost in immunotherapy patients. No associations between ascorbate status and immune-related adverse events or tumour response or overall survival were apparent.

## 1. Introduction

New Zealand has amongst the highest incidence of, and mortality rates from, melanoma in the world (35.8/100,000 and 2.7/100,000, respectively) [1,2,3,4]. Treatment is determined by stage; early (local or regional) disease is treated by surgical resection. The treatment of advanced disease has been revolutionised by immunotherapy (immune checkpoint inhibitors) and BRAF/MEK tyrosine kinase inhibitors [5,6].

Immune checkpoint inhibitors act by preventing checkpoint proteins on immune cells (T cells) from binding with their partner proteins on cancer cells, thus promoting effective anticancer immunity. In 2016, the New Zealand Pharmaceutical Management Agency (Pharmac) funded immune checkpoint inhibitors (nivolumab, pembrolizumab) for advanced disease. Despite substantial improvements in outcome over older treatments, it is known that a substantial proportion of patients do not respond to immunotherapy, and significant numbers of responders and non-responders to treatment experience autoimmune adverse events [7,8]. The development of novel predictors of response and toxicity is a matter of priority.

Response to checkpoint inhibitors has been associated not only with T cells, but also with circulating monocytes [9]. Monocytes are highly plastic innate immune cells that can be divided into three subsets: classical (CD14^+^CD16^−^) monocytes that have heighted pro-inflammatory cytokine production, innate bacterial recognition, migration and phagocytosis capacity, vs. non-classical (CD14^+^CD16^+^) monocytes that patrol the vascular system and are often associated with adhesion and viral and nucleic acid sensing, vs. a third small intermediate subset [10,11,12]. Associations have been reported between outcomes in patients receiving immunotherapy and CD14CD16 expression, but the data are contradictory [13,14,15,16]. The antigen presentation protein major histocompatibility complex cell surface receptor (HLA-DR) is required for the activation of anti-tumour T cells by monocytes. Outcome for patients receiving immunotherapy was associated with CD14^+^HLA-DR expression [15,17,18].

A significant proportion of cancer patients exhibit low plasma vitamin C (ascorbate) levels, to the point of insufficiency [19,20,21,22]. Early immunotherapy studies (using IL-2) reported that patient plasma ascorbate levels dropped to deficient levels (<11 µM) and remained depleted throughout treatment [23,24]. Low plasma ascorbate often correlates with reduced tissue levels of ascorbate, including low immune cell ascorbate concentrations [25], and this could affect immune cell function [26]. Studies have also shown lower levels of ascorbate in circulating immune cells of cancer patients compared to healthy controls [21,25]. The status of ascorbate in patients receiving checkpoint inhibitors is not well characterised.

Ascorbate deficiency in patients with cancer has consequences beyond that of a simple antioxidant deficit. Ascorbate regulates the activity of DNA demethylases (including ten-eleven translocation, TETs), which regulate epigenetic functions [27,28], and modulates the activity of hydroxylases that control response to hypoxia [29]. These enzymes are part of the superfamily of 2-oxoglutarate- and ferrous iron-containing dioxygenases (2-OGDDs) [29]. Previously, we posited that ascorbate affects the functioning of immune cells via these enzymes, and our recent in vitro data showed ascorbate-mediated protein and gene expression changes in cancer-stimulated monocytes in response to ascorbate [26]. Here, we aim to expand this investigation to patient samples.

Our previous study in patients with cancer showed that patients treated with chemotherapy had lower plasma ascorbate levels than those about to undergo surgery to remove their cancer, suggesting that chemotherapy may induce hypovitaminosis C [19]. This provided the rationale for studying the plasma and immune cell ascorbate status of patients undergoing immunotherapy, and for investigating the impact of ascorbate status on monocyte phenotype, TET activity and patient response to immune checkpoint inhibitors. We proposed that ascorbate could modulate monocyte function via TET enzyme activity, leading to changes in DNA methylation, and that this may be associated with patient response to immunotherapy.

## 2. Results

### 2.1. Participant Cohorts

The immunotherapy cohort consisted of 63 patients (Table 1). Most patients in this cohort received pembrolizumab (90%), with 10% receiving nivolumab or other immunotherapies or combinations. This cohort was compared with patients undergoing chemotherapy (*n* = 103) or prior to surgery for cancer (*n* = 82) [19], or healthy, age-matched controls (*n* = 16). Most participants were over 50 years of age, with 54% of the immunotherapy cohort being male, compared to 1/3 of the other two cancer cohorts (Table 1). All patients in the immunotherapy cohort identified as European, compared to the other two cancer cohorts where 11–12% identified as Māori or Pacifica, a percentage representative of the population of the area. All patients in the immunotherapy group had metastatic melanoma, while about half of the other two cohorts had colon or breast cancer, and the remainder had other non-melanoma cancers (Table 1). Patients in the immunotherapy group had received between 1 and 106 rounds of immunotherapy over 0–5 years by the end of 2021.

**Figure 1:**
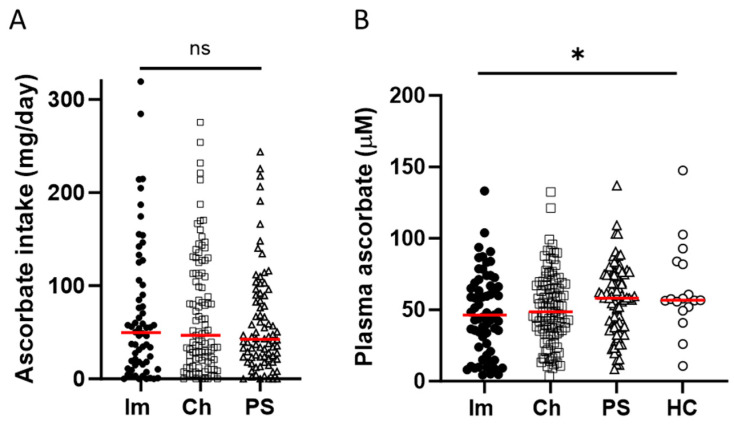
Ascorbate intake and plasma concentrations in patients with cancer and healthy controls. The cohorts included patients receiving immunotherapy (Im) or chemotherapy (Ch), or patients prior to their surgery to remove cancer (PS), or healthy controls (HC). (**A**) Estimated daily intake of ascorbate according to 24 h dietary recall, and (**B**) plasma concentrations of ascorbate, measured by HPLC-EC. Im *n* = 63 filled circles, Ch *n* = 103 open squares, PS *n* = 82 open triangles, HC *n* = 16 open circles, compared by one-way ANOVA, * *p* ≤ 0.05, median shown in red. ns—not significant.

Ascorbate intake, assessed from a 24 h dietary recall, was similar across the three cancer cohorts, with about half of the participants consuming less than the New Zealand recommended daily intake (RDI) of 45 mg ascorbate (Table 1). Average daily intake for the immunotherapy cohort was 69.8 (± 9.3) mg, compared to 68.6 (± 6.3) mg and 60.3 (± 6.2) mg for the chemotherapy and presurgical cohorts, respectively (mean ± SEM, Figure 1). Plasma ascorbate levels varied between the four cohorts, with 24% of the immunotherapy cohort being either marginally ascorbate-deficient (11–23 μM) or deficient (<11 μM), compared to 6–16% for the other cohorts (Table 1). Average plasma ascorbate concentrations were lower in the immunotherapy cohort and chemotherapy cohorts (47.8 ± 3.6 μM and 51.0 ± 2.5 μM) compared to the presurgical cohort (56.8 ± 2.7 μM) and the healthy control cohort (62.3 ± 7.8 μM) (mean ± SEM, *p* ≤ 0.05, Figure 1).

A year after their initial blood draw, ascorbate intake and plasma levels were assessed again in a subset of 16 patients receiving immunotherapy. There was no difference in the average intake or plasma level of ascorbate over this time period (Figure S1A,B). Similarly, a shorter-term assessment conducted over 10 weeks and three clinic visits by patients receiving chemotherapy (*n* = 43) showed that neither ascorbate intake nor plasma levels changed significantly over the 10-week period sampled (Figure S1C,D). We also assessed whether there was any relationship between the number of immunotherapy doses received and the patient’s plasma ascorbate concentration (Figure S2). There was no correlation (Pearson r = 0.053, *p* = 0.74, median no. doses 13, *n* = 41), indicating that the length of treatment did not affect ascorbate status.

### 2.2. PBMC Characterisation of Immunotherapy Cohort

**Figure 2:**
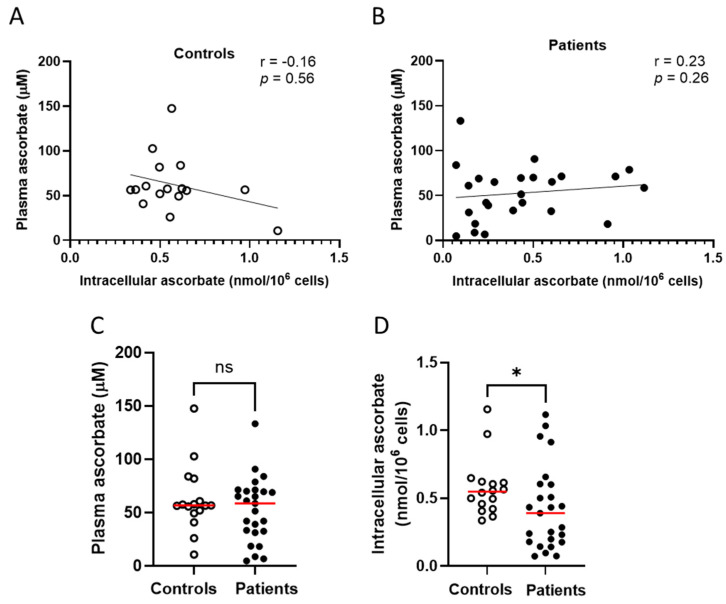
Ascorbate levels in healthy controls and patients with cancer receiving immunotherapy. No relationship between plasma ascorbate and intracellular ascorbate in (**A**) healthy controls or (**B**) patients receiving immunotherapy; Pearson correlation. (**C**) Comparison of plasma levels between patients and controls. (**D**) Comparison of intracellular ascorbate levels between patients and controls; Mann–Whitney test. Intracellular ascorbate was measured by HPLC-EC in peripheral blood-derived mononuclear cells. Patients with metastatic melanoma receiving immunotherapy *n* = 25, healthy controls *n* = 16; ns—not significant, * *p* ≤ 0.05; median shown in red.

Intracellular ascorbate was measured in PBMCs from 25 patients from the immunotherapy cohort, and compared to the control cohort. Intracellular ascorbate in the immunotherapy cohort varied from 0.07 to 1.1 nmol per million cells (mean 0.43 ± 0.06 nmol/10^6^ cells), with plasma levels ranging from 4.8 to 133 μM, with poor correlation between these two measures (Figure 2A). There was also no correlation between plasma and intracellular ascorbate levels in healthy controls (mean 0.58 ± 0.05 nmol/10^6^ cells, Figure 2B), and there was no difference in plasma ascorbate levels between controls and patients (Figure 2C). Intracellular ascorbate was significantly higher in healthy controls compared to immunotherapy patients (Figure 2D).

**Figure 3:**
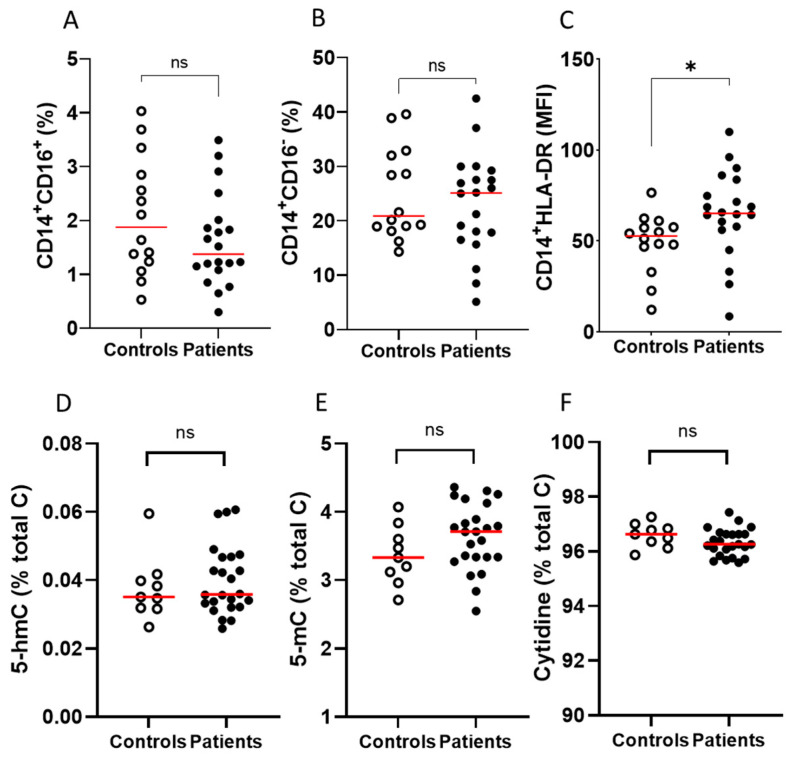
Immune cell and epigenetic markers on PBMCs from patients with metastatic melanoma receiving immunotherapy and healthy volunteers. Comparisons between controls and patients with respect to frequency of (**A**) CD14^+^CD16^+^, (**B**) CD14^+^CD16^−^ and (**C**) HLADR mean fluorescence intensity (MFI). Surface markers were measured by flow cytometry; patients with metastatic melanoma receiving immunotherapy *n* = 20, healthy controls *n* = 14. Comparison between PBMCs from controls and patients with respect to (**D**) 5-hmC, (**E**) 5-mC and (**F**) cytidine content. Epigenetic markers were measured by mass spectrometry; patients with metastatic melanoma receiving immunotherapy *n* = 20, healthy controls *n* = 9; ns—not significant, * *p* ≤ 0.05; median shown in red.

The frequency of non-classical (CD14^+^CD16^+^) and classical (CD14^+^CD16^−^) monocyte populations, and the expression of HLA-DR on CD14^+^ cells in PBMCs, were measured in a subset of patients receiving immunotherapy and healthy controls. PBMCs from the immunotherapy cohort contained similar frequencies of non-classical (CD14^+^CD16^+^) and classical monocytes (CD14^+^CD16^−^) to PBMCs from healthy controls (Figure 3A,B). Interestingly, HLA-DR expression was significantly higher (*p* ≤ 0.05) in the immunotherapy cohort compared to the healthy controls (Figure 3C). There were no differences in epigenetic markers (5-hmC, 5-mC or cytidine) between controls and patients (Figure 3D–F).

**Figure 4:**
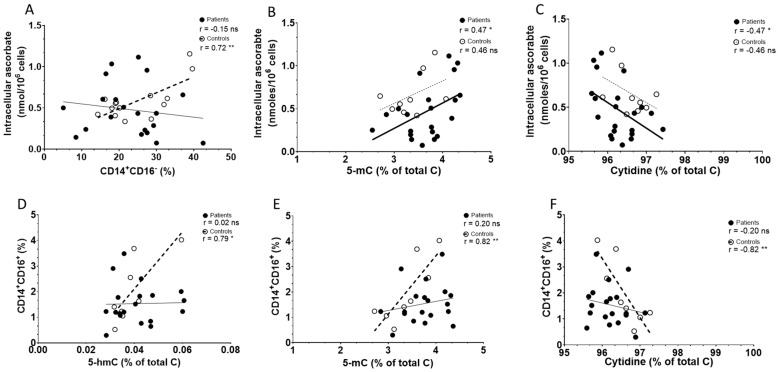
Associations between ascorbate and immune cell and epigenetic markers in PBMCs from controls and patients with melanoma. Correlations between intracellular ascorbate and (**A**) percentage of classical monocytes (CD14^+^16^−^), (**B**) 5-mC and (**C**) cytidine. Correlations between percentage of non-classical monocytes (CD14^+^CD16^+^) and epigenetic markers (**D**) 5-hmC, (**E**) 5-mC and (**F**) cytidine. Patient data are shown as filled circles with solid lines, and those of controls as open circles with dashed lines; bold lines indicate significance. Relationships were tested using Pearson’s correlation; ns—not significant, significance * *p* < 0.05, ** *p* < 0.01.

As ascorbate may mechanistically influence innate immune cells [26,30], we investigated relationships between intracellular ascorbate concentration and the presence of epigenetic markers, such as 5-mC and the TET product 5-hmC, against the frequency of monocyte subsets (CD14^+^CD16^+^, CD14^+^CD16^−^, HLA-DR) in the cohorts (Table 2). Intracellular ascorbate was strongly associated with classical monocyte frequency (CD14^+^CD16^−^) in controls but not patients (Figure 4A), and no associations were evident with non-classical monocytes. Intracellular ascorbate was positively associated with 5-mC and negatively with total cytidine in patients, but not controls, with no associations observed with 5-hmC (Figure 4B,C). There was a weak relationship between ascorbate and HLA-DR expression in patients only, trending towards significance (*p* = 0.06). There was also a weak correlation in controls only between 5-hmC and HLA-DR (*p* = 0.07). The levels of 5-hmC and 5-mC in PBMCs correlated strongly with non-classical monocytes in controls only, not patients (Figure 4D,E), and no other relationships with 5-hmC/5-mC and monocyte subsets were evident. Cytidine correlated negatively with non-classical monocytes in controls (Figure 4F), with no other relationships in controls or patients evident (Table 2).

### 2.3. Treatment Response of Patients Receiving Immunotherapy

**Figure 5:**
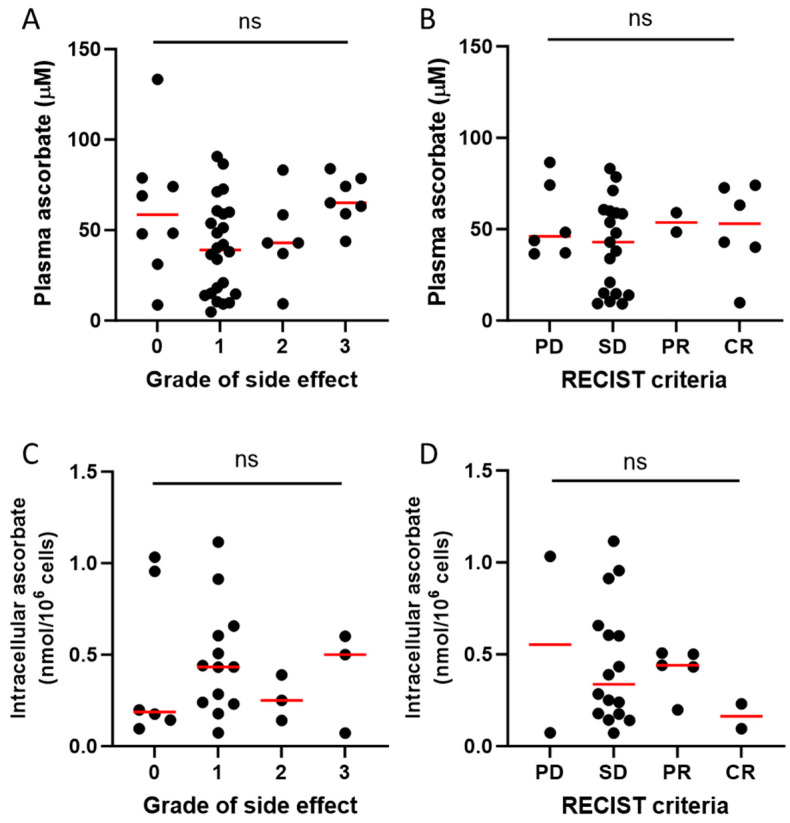
Relationship of ascorbate status and patient or tumour response to immunotherapy. Plasma ascorbate levels vs. (**A**) grade of side effects in patients with metastatic melanoma receiving immunotherapy, and (**B**) tumour response based on RECIST criteria. Intracellular ascorbate in PBMCs vs. (**C**) grade of side effects and (**D**) RECIST criteria. PD—progressive disease, SD—stable disease, PR—partial response, CR—complete response; plasma data for side effects available from *n* = 45 and for RECIST for *n* = 33 patients; intracellular ascorbate data available for *n* = 25 patients; ns – not significant; median shown in red.

Immunotherapy commonly induces immune-related adverse events, some of which can be life-threatening [8]. To ascertain whether ascorbate status affected these adverse events, we compared plasma ascorbate status against the severity of side effects (Figure 5A). Most patients in this study had grade 1 side effects as reported in the medical notes, with 18% having no reported side effects, and there was no relationship between plasma ascorbate levels and severity of side effects in response to immunotherapy (Figure 5A). We saw no association between plasma ascorbate levels and reported tumour response based on RECIST criteria (Figure 5B). There was also no difference in side effects or tumour response according to the intracellular PBMC ascorbate levels (Figure 5C,D).

**Figure 6:**
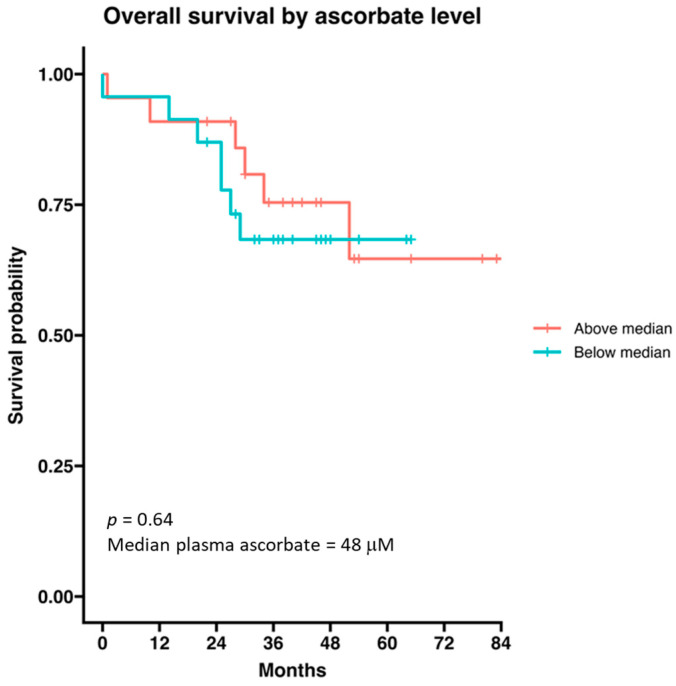
Survival probability of patients with metastatic melanoma receiving immunotherapy according to ascorbate status. The cohort was divided by median ascorbate (48 μM). Data are presented as a Kaplan–Meier curve; *n* = 44.

Median survival in the whole treatment group was not reached (*n* = 44). The median survival in those with disease progression was 27 months (data not shown). Survival of the immunotherapy cohort was not influenced by age, sex or ascorbate as a continuous variable. When the cohort was divided into above and below median plasma ascorbate levels (48 μM), we saw no difference in overall survival according to ascorbate status (Figure 6).

## 3. Discussion

Our data on patients with metastatic melanoma undergoing immune checkpoint therapy showed that a quarter of the cohort had hypovitaminosis C, with plasma ascorbate levels in this cohort significantly lower than those from other cancer patients or healthy controls. PBMC ascorbate levels were also significantly lower than those of healthy controls and there was limited correlation between plasma and immune cell ascorbate. Blood mononuclear cells from the immunotherapy cohort contained similar frequencies of non-classical and classical monocytes to, but exhibited higher levels of HLA-DR expression than, those from healthy controls. Predominately in healthy controls, methylation markers and intracellular ascorbate concentration were correlated with monocyte subset frequency, a relationship lost in immunotherapy patients. In this small patient cohort, no associations between ascorbate status and immune-related adverse events or tumour response or overall survival were apparent.

Ascorbate intake across the three cancer cohorts was similar, with about half consuming less than the NZ recommended daily intake of 45 mg ascorbate, one of the lowest RDIs in the world (90 mg/day in the UK, Europe and the US) [31]. Yet plasma levels of ascorbate were significantly lower in patients undergoing immuno- or chemotherapy compared to patients prior to cancer surgery or healthy controls, confirming earlier reports [19,22,24]. This may indicate either that these treatments, or that advanced/metastatic disease, affect ascorbate turnover. This aligns with findings for other chronic or severe diseases, such as diabetes and burns [32]. Intracellular ascorbate was particularly low in PBMCs from immunotherapy patients. This may be suggestive of PBMC activation to tumour neoantigens and ascorbate’s possible role in underlying mechanisms. Ascorbate’s involvement in differentiation, reactive oxygen species production, phagocytosis, chemotaxis and epigenetic enzyme activity in activated immune cells may result in high turnover of the vitamin [30]. It is important, however, to point out that our data on cellular ascorbate, immune cell markers and epigenetic markers are derived from a small cohort of patients and controls. Further studies in larger cohorts are required to confirm our findings.

The lack of correlation between plasma and PBMC ascorbate content was unexpected, as others have reported an association, although a lack of correlation has been reported between normal and tumour tissue from the same patient [33,34,35]. Our data may reflect that the time-delay in assessing intracellular ascorbate, necessitated by immune cell isolation procedures, may have resulted in loss of ascorbate. We limited this possibility by using a quick isolation kit that involves density separation with the centrifugation brake on, as opposed to no brake, and by reducing all samples using TCEP to obtain total ascorbate [32]. As the lack of association was also seen in samples from healthy controls, it likely did not arise as a result of the cancer or therapy.

Studies have demonstrated that higher classical monocyte frequencies were associated with reduced overall survival and tumour resistance, while non-classical monocytes were associated with improved progression-free survival in patients receiving immunotherapy [13,14,15]. In contrast, it has also been reported that upregulation of both classical and non-classical monocytes correlated with anti-PD1 therapy response [16]. As we only had monocyte data for a total of 20 patients, the survival analysis was underpowered, and we could not confirm survival associations with monocyte subsets.

CD14^+^HLA-DR expression has been reported as a predictor of immunotherapy response [17], with increased expression suggesting activation and greater presentation of tumour neoantigens to reactivated T cells, favouring positive therapy outcomes [17]. The increased expression of HLA-DR in our immunotherapy cohort is indicative of possible monocyte activation by the tumour and may contribute towards patient retention of the immunotherapy. As many of the patients within this study have received immunotherapy for extended periods, we may have indirectly selected patients with greater HLA-DR expression. This is particularly plausible as CD14^+^HLA-DR ^low/−^ phenotypes are typically associated with therapy-resistant tumours, causing therapy discontinuation [18]. Interestingly, within the patient cohort, HLA-DR expression was positively correlated with intracellular ascorbate concentration, a relationship that was not seen in healthy controls.

The associations between non-classical monocyte frequency and the presence of 5-hmC, 5-mC and cytidine were strong in healthy controls, making the loss of this relationship in the immunotherapy cohort unexpected. The reasons for this are unclear, but may indicate differentiation of non-classical monocytes from classical phenotypes [11,36]. We hypothesise that immunotherapy, or prolonged immune response brought on by checkpoint inhibitors, may disrupt epigenetic modifications involved with differentiation [12,37]. Interestingly, classical monocyte frequency was positively associated with intracellular ascorbate concentration in healthy controls, but lost in the immunotherapy cohort, suggesting that ascorbate may have an active role in the maturation and/or release of classical monocytes from bone marrow only in healthy controls [34,36]. It is noteworthy that the methylation status is measured in all PBMCs whereas CD14^+^CD16^+^ cells only make up a very small proportion. Further work is required to clarify any relationships and possible mechanisms.

We saw no associations between ascorbate and 5-hmC in PBMCs from either the healthy or cancer cohorts. The epigenetic marker 5-hmC is the first step in demethylation via ascorbate-dependent dioxygenase enzymes (TETs) and de-repression of global gene expression [27]. How ascorbate influences TET activity in the context of cancer, and patients receiving immunotherapy, remains unclear. Our data showed that 5-mC levels strongly correlated with intracellular ascorbate in patients, but not controls. The epigenetic marker 5-mC is associated with repression of gene expression, with our data indicating that patients’ PBMCs showed repressed gene expression, possibly associated with terminal differentiation.

Immunotherapy patients were recruited at any point along their treatment journey, with some having recently started therapy whereas others had been treated for several years. A subgroup of the cohort was followed for more than a year, and another over two or more appointments, but median intake and plasma ascorbate levels did not change over these periods, indicating that dietary habits did not change and that ongoing therapy did not adversely affect their ascorbate status. As NZ only continues to fund immunotherapy for those showing response, our cohort was likely enriched with patients responding to immunotherapy.

Our data indicated that neither recorded severity of side effects (immune-related adverse events) nor extent of tumour response (based on RECIST criteria) were associated with plasma ascorbate levels. We also saw no survival differences when patients were stratified by ascorbate status. This is not entirely unexpected, as although the molecular pathways governed by ascorbate are vital for immune cell maturation and function via 2-OGDD enzyme function [29], each patient is very complex, and numerous intrinsic and extrinsic effects govern response. Side effects in our cohort were common, with most at the low severity grade. This may reflect that patients who have more severe adverse events do not continue treatment and that those with progressive disease will no longer have their treatment funded, and thus likely reflects a bias for responders, as discussed above.

Melanomas are infiltrated by numerous immune cell types, besides monocytes and T cells, that may modulate response to checkpoint inhibition, such as dendritic cells, neutrophils and mast cells [38,39]. As the PBMCs could have contained a proportion of these cell types, they may have contributed to the cellular ascorbate content.

Whether and how the ascorbate status of patients undergoing immunotherapy has clinical implications remains to be determined. Clinical trials using ascorbate intervention in patients with melanoma have not yet been reported, but results from animal studies are encouraging. Melanomas grew more slowly and were rejected more effectively [40,41] in ascorbate-treated mice, and immunotherapy treatment against melanoma tumours was enhanced by ascorbate injections in mice [42,43].

Our observational study had several additional limitations. We were constrained by the availability of patient samples, with most individuals providing a single sample at one point during their immunotherapy schedule. An additional limitation was the use of unrelated healthy controls, although this was mitigated to an extent by choosing age-matched individuals.

Sophisticated techniques, such as single cell analyses and DNA methylation microarray epigenotyping, are needed to confirm the role of ascorbate as a cofactor of dioxygenase enzymes driving the associations between ascorbate and immune cell markers and epigenetic modifications. To establish whether there is an effect of therapy on ascorbate measures and whether ascorbate status modifies treatment response, samples before and after therapy should be compared in future.

## 4. Materials and Methods

### 4.1. Ethics, Consent and Eligibility

This study was approved by the New Zealand Health and Disability Ethics Committee (18/STH/223) with amendments AM01-AM04. Patients were recruited at Christchurch Hospital, Te Whatu Ora Waitaha, Aotearoa/New Zealand, and age-matched healthy controls were recruited at the University of Otago, Christchurch. Participants gave informed consent for a blood draw of 5–10 mL via venipuncture, for administration of a brief dietary questionnaire and for access to relevant medical information (patients only). Participants’ ethnicity was self-declared using the New Zealand census questions. Participants were aged >18, with a confirmed diagnosis of cancer (patients only). Healthy controls had no known current diagnosis of cancer. Only participants who had been fasting prior to the blood draw, defined as no consumption of ascorbate in the previous 8 h, were included in this study.

### 4.2. Study Participants

Participants were recruited between November 2018 and January 2022. Patients in the immunotherapy cohort all had a diagnosis of metastatic melanoma and were recruited from the Oncology Service, Christchurch Hospital. Patients in the immunotherapy cohort were all partway through their treatment schedule, with at least one prior treatment. The chemotherapy cohort included patients with various types of cancers, with 69 of *n* = 103 included in a previous study [19]. The presurgical cohort (*n* = 82) included patients with a confirmed cancer diagnosis and who were all part of a previous study [19]. Healthy volunteers were recruited as controls (*n* = 16).

All *n* = 63 patients receiving immunotherapy provided plasma ascorbate data, *n* = 25 had intracellular ascorbate data, and *n* = 20 had monocyte subset and DNA methylation data, with follow-up data on *n* = 45 for adverse reactions, *n* = 33 for treatment response and *n* = 44 for overall survival. Of the healthy control cohort, all *n* = 16 had plasma and intracellular ascorbate data, *n* = 14 had monocyte subset data and *n* = 9 had DNA methylation data.

### 4.3. Patient Follow-Up

Patients with metastatic melanoma receiving immunotherapy were followed up for ~4 years after the blood draw and up to 7 years after diagnosis of metastatic melanoma. Side effects, tumour response and overall survival were collected from clinical records. The severity of immune-mediated adverse reactions over their treatment course was assessed, with the highest reported grade documented. The grade was determined from the clinical record using the CTCAE criteria (Version 5), where 0 represents no effect reported and 4 is the most severe [44]. Best response to treatment was extracted from the clinical record and classified as progressive disease (PD), stable disease (SD), partial response (PR) or complete response (CR) [45]. Follow-up for survival was carried out for up to 7 years (until November 2023). Survival was calculated from the date of first treatment until the date of death.

### 4.4. Materials

Unless specified, all chemicals were from Sigma-Aldrich (St. Louis, MO, USA).

### 4.5. Blood Sample and Plasma Collection

Peripheral blood was drawn by experienced phlebotomists, collected into 10 mL sodium heparin vacutainer tubes (Becton Dickinson, Auckland, New Zealand) and placed on ice. To preserve ascorbate content, all samples were processed within 1 h of collection. For plasma collection, samples were centrifuged at 4 °C, and plasma was collected and mixed with ice-cold perchloric acid (540 mM PCA) with diethylene-triaminepenta-acetic acid (100 μM DTPA). Samples were centrifuged to remove protein flocculants and the cleared plasma was stored at −80 °C until analysis.

### 4.6. Isolation of Blood Cells

Peripheral blood-derived mononuclear cells (PBMCs) were harvested from the blood using lymphoprep density gradient centrifugation in SepMate PBMC Isolation tubes (StemCell Technologies, Tullamarine, Australia) and counted. One million PBMCs each were required for ascorbate, mass spectrometry and flow cytometry analysis. For intracellular ascorbate analysis, PBMCs were lysed by addition of PCA with DTPA, and frozen at −80 °C until ascorbate analysis. For flow cytometry, PBMCs were dispersed in RPMI 1640 + GlutaMax buffer (Life Technologies^TM^, Carlsbad, CA, USA) and processed for antibody staining. For mass spectrometry, DNA was extracted with a kit (NucleoSpin Tissue, Macherey-Nagel, Allentown, PA, USA) and stored at −80 °C.

### 4.7. Ascorbate Analysis

Average daily intake of ascorbate was estimated from the dietary questionnaire, as described previously [19]. Ascorbate content of plasma and PBMCs was measured using reverse-phase high-performance liquid chromatography with electrochemical detection (HPLC-EC). Briefly, to obtain total ascorbate content, samples were reduced with Tris-(2-carboxyethyl)-phosphine (TCEP), and then diluted in PCA with DTPA, and samples were separated by HPLC-EC [19]. For every run, a standard curve of sodium-L-ascorbate was freshly prepared. Plasma ascorbate concentration is expressed as μM and intracellular ascorbate is expressed as nmol ascorbate per one million cells.

### 4.8. Flow Cytometry

PBMCs concentrated to 5 × 10^6^ cells/mL were stained with fluorescent antibodies directed against the surface markers CD14, CD16 and HLA-DR (CD14:PerCP, CD16:FITC, HLA-DR:PE) or IgG controls (BD Biosciences, Thermo Fisher Scientific, Sunnyvale, CA, USA). Fluorescent cells were measured by flow cytometry (Beckman Coulter, Cytomics FC 500 MPL, Miami, FL, USA). After compensation, the mean fluorescence intensity (MFI), frequency of cells positively stained and resultant populations based on the combination of markers were analysed using CXP Analysis (Beckman Coulter, Brea, CA, USA).

### 4.9. Mass Spectrometry

Nucleotides were liberated from the harvested DNA using a nucleotide digestion kit (New England BioLabs, Ipswich, MA, USA). Samples were spiked with 85 fmoles of cytidine, 5 fmoles of 5-methylcytidine (5-mC) and 0.13 fmoles of 5-hydroxymethylcytidine (5-hmC) heavy isotopes. The cytidine species were measured using mass spectrometry (QTRAP^®^6500) as the percentage of total cytidine species, as described previously [46].

### 4.10. Statistical Analyses

Data were analysed using GraphPad Prism V.10. Normality of data was assessed using the D’Agostino and Pearson test. Categorical variables were compared by unpaired *t*-tests, the Mann–Whitney test or ANOVA followed by Tukey’s post hoc tests. The relationships between variables were assessed using Pearson correlation coefficients or Spearman correlation for normally or non-normally distributed data, respectively. Survival analysis was performed using R (Version 4.2.0 (22 April 2022)) and the Survival and Survminer packages [47]. All *p* values are two-tailed, unpaired and with statistical significance set at *p* < 0.05.

## 5. Conclusions

This study demonstrates that patients with metastatic melanoma receiving immunotherapy have particularly low plasma and PBMC ascorbate levels. This indicates a need for ascorbate supplementation, although patient ascorbate status did not align with response to therapy. Associations between non-classical monocytes and cytidine species in PBMCs were strong in healthy controls but lost in patients receiving immunotherapy. These complex findings require further investigation in much larger cohorts to ascertain their clinical implications.

## Figures and Tables

**Table 1:** Participant demographics and information.

		Immunotherapy	Chemotherapy	Presurgical *	Healthy Control
Total		*n = 63*	*n = 103*	*n = 82*	*n = 16*
Age	≤50 years	7 (11%)	32 (31%)	15 (18%)	5 (31%)
Sex	Female	29 (46%)	69 (67%)	49 (60%)	9 (56%)
Ethnicity	European	63 (100%)	88 (85%)	71 (87%)	15 (94%)
	Māori/Pacifica	0 (0%)	12 (12%)	9 (11%)	1 (6%)
	Other	0 (0%)	3 (3%)	2 (2%)	0 (0%)
Cancer surgery		n/d	n/d	82 (100%)	n/a
Chemotherapy		n/d	103 (100%)	n/d	n/a
Immunotherapy	Pembrolizumab	57 (90.5%)	n/a	n/a	n/a
	Nivolumab	4 (6.3%)	n/a	n/a	n/a
	Combination	2 (3.2%)	n/a	n/a	n/a
Cancer type	Melanoma	63 (100%)	0 (0%)	1 (1%)	n/a
	Colon, breast	0 (0%)	52 (50%)	45 (55%)	n/a
	Other	0 (0%)	51 (50%)	36 (44%)	n/a
Ascorbate intake	≤45 mg/day	28 (45%)	51 (50%)	43 (52%)	n/d
Plasma ascorbate	≤23 μmol	15 (24%)	16 (16%)	8 (9.8%)	1 (6%)

* Blood samples taken prior to surgery, n/a—not applicable, n/d—not determined.

**Table 2:** Relationships in PBMCs from controls and patients with intracellular ascorbate and immune cell and epigenetic markers.

		Controls		Patients	
Correlates		r ^a^	*p* ^b^	r ^a^	*p* ^b^
Intracellular ascorbate ^c^	5-hmC ^e^	0.27	0.49	0.10	0.66
	5-mC ^e^	0.46	0.22	**0.47**	**0.03**
	Cytidine ^e^	−0.46	0.22	**−0.47**	**0.03**
	CD14^+^CD16^+ g^	0.21	0.47	0.03	0.90
	CD14^+^CD16^− g^	**0.72**	**0.004**	−0.15	0.52
	HLA-DR (CD14^+^) ^f^	−0.14	0.63	0.43	0.06
Plasma ascorbate ^d^	5-hmC ^e^	−0.03	0.95	−0.37	0.12
	5-mC ^e^	−0.12	0.76	0.04	0.88
	Cytidine ^e^	0.12	0.76	−0.03	0.91
	CD14^+^CD16^+ g^	−0.02	0.95	0.16	0.53
	CD14^+^CD16^− g^	<−0.01	>0.99	0.37	0.14
	HLA-DR (CD14^+^) ^f^	0.22	0.45	0.04	0.89
5-hmC ^e^	CD14^+^CD16^− g^	**0.79**	**0.02**	0.02	0.92
	CD14^+^CD16^− g^	0.39	0.34	−0.11	0.64
	HLA-DR (CD14^+^) ^f^	0.68	0.07	0.07	0.78
5-mC ^e^	CD14^+^CD16^+ g^	**0.82**	**0.01**	0.20	0.41
	CD14^+^CD16^− g^	0.51	0.20	0.22	0.37
	HLA-DR (CD14^+^) ^f^	0.09	0.83	0.07	0.76
Cytidine ^e^	CD14^+^CD16^+ g^	**−0.82**	**0.01**	−0.20	0.42
	CD14^+^CD16^− g^	−0.50	0.20	−0.21	0.38
	HLA-DR (CD14^+^) ^f^	−0.10	0.81	−0.08	0.75

^a^ Pearson’s r, ^b^ significant correlations are shown in bold, ^c^ nmol/10^6^ cells, ^d^ µM, ^e^ % total cytidine, ^f^ MFI within CD14^+^ cells, ^g^ frequency of total PBMC; non-classical monocytes (CD14^+^CD16^+^), classical monocytes (CD14^+^CD16^−^).

## Data Availability

All data are available within this manuscript with the exception of data unavailable due to privacy or ethical restrictions.

## Supplementary Materials

The following supporting information can be downloaded at https://www.mdpi.com/article/10.3390/epigenomes8020017/s1: Figure S1: Ascorbate intake and plasma levels of patients with metastatic melanoma receiving immunotherapy followed over time. Figure S2: Relationship between the total number of immunotherapy doses received at the time of blood draw and plasma ascorbate concentration in patients with metastatic melanoma.

## References

[1] Dzwierzynski W.W. (2021). Melanoma Risk Factors and Prevention. Clin. Plast. Surg..

[2] Cancer Society Melanoma Survival Rates|Melanoma Survival Statistics. https://www.cancer.org/cancer/melanoma-skin-cancer/detection-diagnosis-staging/survival-rates-for-melanoma-skin-cancer-by-stage.html.

[3] Ahmadi F., Karamitanha F., Ramezanpour A. (2022). Clustering trends of melanoma incidence and mortality: A worldwide assessment from 1995 to 2019. Australas. J. Dermatol..

[4] Huang J., Chan S.C., Ko S., Lok V., Zhang L., Lin X., Lucero-Prisno D.E., Xu W., Zheng Z.J., Elcarte E. (2023). Global incidence, mortality, risk factors and trends of melanoma: A systematic analysis of registries. Am. J. Clin. Dermatol..

[5] Villani A., Scalvenzi M., Micali G., Lacarrubba F., Fornaro L., Martora F., Potestio L. (2023). Management of Advanced Invasive Melanoma: New Strategies. Adv. Ther..

[6] Board R., Smittenaar R., Lawton S., Liu H., Juwa B., Chao D., Corrie P. (2021). Metastatic melanoma patient outcomes since introduction of immune checkpoint inhibitors in England between 2014 and 2018. Int. J. Cancer.

[7] Hamid O., Robert C., Daud A., Hodi F.S., Hwu W.J., Kefford R., Wolchok J.D., Hersey P., Joseph R., Weber J.S. (2019). Five-year survival outcomes for patients with advanced melanoma treated with pembrolizumab in KEYNOTE-001. Ann. Oncol..

[8] Waldman A.D., Fritz J.M., Lenardo M.J. (2020). A guide to cancer immunotherapy: From T cell basic science to clinical practice. Nat. Rev. Immunol..

[9] Parikh K., Kumar A., Ahmed J., Anwar A., Puccio C., Chun H., Fanucchi M., Lim S.H. (2018). Peripheral monocytes and neutrophils predict response to immune checkpoint inhibitors in patients with metastatic non-small cell lung cancer. Cancer Immunol. Immunother..

[10] Gren S.T., Rasmussen T.B., Janciauskiene S., Håkansson K., Gerwien J.G., Grip O. (2015). A single-cell gene-expression profile reveals inter-cellular heterogeneity within human monocyte subsets. PLoS ONE.

[11] Kapellos T.S., Bonaguro L., Gemünd I., Reusch N., Saglam A., Hinkley E.R., Schultze J.L. (2019). Human Monocyte Subsets and Phenotypes in Major Chronic Inflammatory Diseases. Front. Immunol..

[12] Thomas G., Tacke R., Hedrick C.C., Hanna R.N. (2015). Nonclassical patrolling monocyte function in the vasculature. Arterioscler. Thromb. Vasc. Biol..

[13] Ohkuma R., Fujimoto Y., Ieguchi K., Onishi N., Watanabe M., Takayanagi D., Goshima T., Horiike A., Hamada K., Ariizu-mi H. (2023). Monocyte subsets associated with the efficacy of anti PD 1 antibody monotherapy. Oncol. Lett..

[14] Rodriguez B.L., Chen L., Li Y., Miao S., Peng D.H., Fradette J.J., Diao L., Konen J.M., Alvarez F.R.R., Solis L.M. (2023). Targeting immunosuppressive Ly6C+ classical monocytes reverses anti-PD-1/CTLA-4 immunotherapy resistance. Front. Immunol..

[15] Pico de Coaña Y., Wolodarski M., van der Haar Àvila I., Nakajima T., Rentouli S., Lundqvist A., Masucci G., Hansson J., Kiessling R. (2020). PD-1 checkpoint blockade in advanced melanoma patients: NK cells, monocytic subsets and host PD-L1 expression as predictive biomarker candidates. Oncoimmunology.

[16] Olingy C., Alimadadi A., Araujo D.J., Barry D., Gutierrez N.A., Werbin M.H., Arriola E., Patel S.P., Ottensmeier C.H., Dinh H.Q. (2022). CD33 expression on peripheral blood monocytes predicts efficacy of anti-PD-1 immunotherapy against non-small cell lung cancer. Front. Immunol..

[17] Krieg C., Nowicka M., Guglietta S., Schindler S., Hartmann F.J., Weber L.M., Dummer R., Robinson M.D., Levesque M.P., Becher B. (2018). High-dimensional single-cell analysis predicts response to anti-PD-1 immunotherapy. Nat. Med..

[18] Mengos A.E., Gastineau D.A., Gustafson M.P. (2019). The CD14+HLA-DRlo/neg Monocyte: An immunosuppressive phenotype that restrains responses to cancer immunotherapy. Front. Immunol..

[19] White R., Nonis M., Pearson J.F., Burgess E., Morrin H.R., Pullar J.M., Spencer E., Vissers M.C.M., Robinson B.A., Dachs G.U. (2020). Low vitamin C status in patients with cancer is associated with patient and tumor characteristics. Nutrients.

[20] Huijskens M.J., Wodzig W.K., Walczak M., Germeraad W.T., Bos G.M. (2016). Ascorbic acid serum levels are reduced in patients with hematological malignancies. Results Immunol..

[21] Anthony H.M., Schorah C.J. (1982). Severe hypovitaminosis C in lung-cancer patients: The utilization of vitamin C in surgical repair and lymphocyte-related host resistance. Br. J. Cancer..

[22] Schleich T., Rodemeister S., Venturelli S., Sinnberg T., Garbe C., and Busch C. (2013). Decreased plasma ascorbate levels in stage IV melanoma patients. Metab. Nutr. Oncol..

[23] Marcus S.L., Dutcher J.P., Paietta E., Ciobanu N., Strauman J., Wiernik P.H., Hutner S.H., Frank O., Baker H. (1987). Severe hypovitaminosis C occurring as the result of adoptive immunotherapy with high-dose interleukin 2 and lymphokine-activated killer cells. Cancer Res..

[24] Marcus S.L., Petrylak D.P., Dutcher J.P., Paietta E., Ciobanu N., Strauman J., Wiernik P.H., Hutner S.H., Frank O.O., Baker H. (1991). Hypovitaminosis C in patients treated with high-dose interleukin 2 and lymphokine-activated killer cells. Am. J. Clin. Nutr..

[25] Basu T.K., Raven R.W., Dickerson J.W., Williams D.C. (1974). Leucocyte ascorbic acid and urinary hydroxyproline levels in patients bearing breast cancer with skeletal metastases. Eur. J. Cancer..

[26] Ang A.D., Vissers M.C.M., Burgess E.R., Currie M.J., Dachs G.U. (2021). Gene and protein expression is altered by ascorbate availability in murine macrophages cultured under tumour-like conditions. Antioxidants.

[27] Yue X., Rao A. (2020). TET family dioxygenases and the TET activator vitamin C in immune responses and cancer. Blood.

[28] Mikkelsen S.U., Gillberg L., Lykkesfeldt J., Grønbæk K. (2021). The role of vitamin C in epigenetic cancer therapy. Free Radic. Biol. Med..

[29] Vissers M.C.M., Das A.B. (2018). Potential Mechanisms of Action for Vitamin C in Cancer: Reviewing the Evidence. Front. Physiol..

[30] Carr A.C., Maggini S. (2017). Vitamin C and immune function. Nutrients.

[31] Institute of Medicine (US) Panel on Dietary Antioxidants and Related Compounds (2000). Dietary Reference Intakes for Vitamin C, Vitamin E, Selenium, and Carotenoids.

[32] Berretta M., Quagliariello V., Maurea N., Di Francia R., Sharifi S., Facchini G., Rinaldi L., Piezzo M., Manuela C., Nunnari G. (2020). Multiple effects of ascorbic acid against chronic diseases: Updated evidence from preclinical and clinical studies. Antioxidants.

[33] Wohlrab C., Vissers M.C.M., Phillips E., Morrin H., Robinson B.A., Dachs G.U. (2018). The association between ascorbate and the hypoxia-inducible factors in human renal cell carcinoma requires a functional von Hippel-Lindau protein. Front. Oncol..

[34] Levine M., Conry-Cantilena C., Wang Y., Welch R.W., Washko P.W., Dhariwal K.R., Park J.B., Lazarev A., Graumlich J.F., King J. (1996). Vitamin C pharmacokinetics in healthy volunteers: Evidence for a recommended dietary allowance. Proc. Natl. Acad. Sci. USA.

[35] Levine M., Wang Y., Padayatty S.J., Morrow J. (2001). A new recommended dietary allowance of vitamin C for healthy young women. Proc. Natl. Acad. Sci. USA.

[36] Patel A.A., Zhang Y., Fullerton J.N., Boelen L., Rongvaux A., Maini A.A., Bigley V., Flavell R.A., Gilroy D.W., Asquith B. (2017). The fate and lifespan of human monocyte subsets in steady state and systemic inflammation. J. Exp. Med..

[37] Bharat A., McQuattie-Pimentel A.C., Budinger G.S. (2017). Non-classical monocytes in tissue injury and cancer. Oncotarget.

[38] Antohe M., Nedelcu R.I., Nichita L., Popp C.G., Cioplea M., Brinzea A., Hodorogea A., Calinescu A., Balaban M., Ion D.A. (2019). Tumor infiltrating lymphocytes: The regulator of melanoma evolution. Oncol. Lett..

[39] Li J., Peng G., Zhu K., Jie X., Xu Y., Rao X., Xu Y., Chen Y., Xing B., Wu G. (2023). PD-1+ mast cell enhanced by PD-1 blocking therapy associated with resistance to immunotherapy. Cancer Immunol. Immunother..

[40] Niessner H., Burkard M., Leischner C., Renner O., Plöger S., Meraz-Torres F., Böcker M., Hirn C., Lauer U.M., Venturelli S. (2022). Therapeutic Efficacy of Pharmacological Ascorbate on Braf Inhibitor Resistant Melanoma Cells In Vitro and In Vivo. Cells.

[41] Campbell E.J., Vissers M.C.M., Dachs G.U. (2016). Ascorbate availability affects tumor implantation-take rate and increases tumor rejection in Gulo(-/-) mice. Hypoxia.

[42] Burkard M., Niessner H., Leischner C., Piotrowsky A., Renner O., Marongiu L., Lauer U.M., Busch C., Sinnberg T., Venturelli S. (2023). High-Dose Ascorbate in Combination with Anti-PD1 Checkpoint Inhibition as Treatment Option for Malignant Melanoma. Cells.

[43] Magrì A., Germano G., Lorenzato A., Lamba S., Chilà R., Montone M., Amodio V., Ceruti T., Sassi F., Arena S. (2020). High-dose vitamin C enhances cancer immunotherapy. Sci. Transl. Med..

[44] (2017). Common Terminology Criteria for Adverse Events Version 5.0. https://ctep.cancer.gov/protocoldevelopment/electronic_applications/docs/ctcae_v5_quick_reference_5x7.pdf.

[45] Eisenhauer E.A., Therasse P., Bogaerts J., Schwartz L.H., Sargent D., Ford R., Dancey J., Arbuck S., Gwyther S., Mooney M. (2009). New response evaluation criteria in solid tumours: Revised RE-CIST guideline (version 1.1). Eur. J. Cancer.

[46] Smith-Díaz C.C., Magon N.J., McKenzie J.L., Hampton M.B., Vissers M.C.M., Das A.B. (2021). Ascorbate inhibits proliferation and promotes myeloid differentiation in TP53-mutant leukemia. Front. Oncol..

[47] Kassambara A., Kosinski M., Biecek P. Drawing Survival Curves Using g‘gplot2’. R Package Version 0.4.9. https://cran.r-project.org/web/packages/survminer/survminer.pdf.

