# Supplementary material for: Blood Vitamin C Levels of Patients Receiving Immunotherapy and Relationship to Monocyte Subtype and Epigenetic Modification
Source: Epigenomes. 2024 Apr 30;8(2):17. doi: 10.3390/epigenomes8020017 (PMC11130941; doi:10.3390/epigenomes8020017)
Supplement: Supplementary file 1 [file epigenomes-08-00017-s001.zip › epigenomes-2895811-supplementary.pdf]

Supplementary Figures for

Blood vitamin C levels of patients receiving immunotherapy and relationship to monocyte subtype and epigenetic modification

by Topham et al

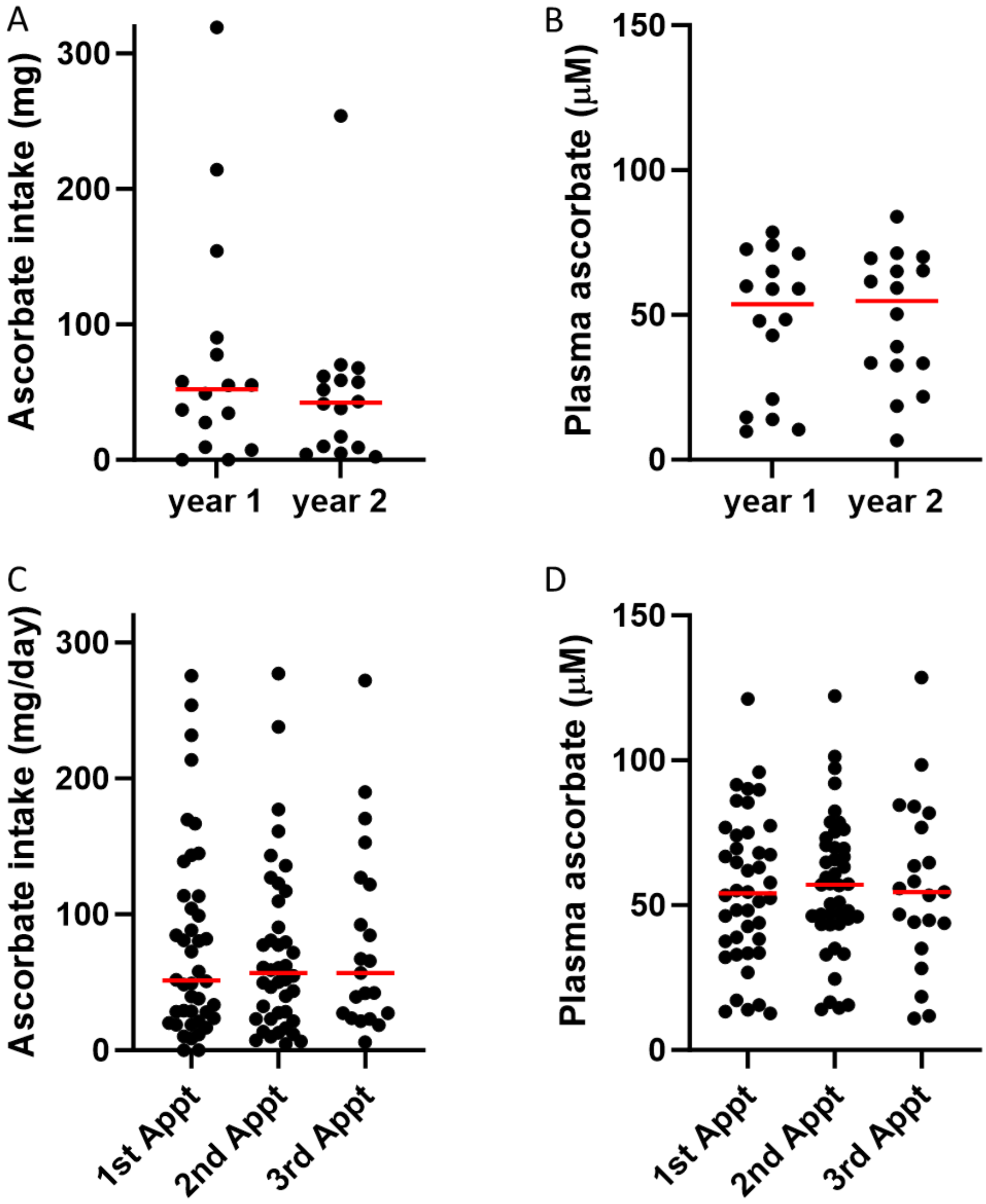

Figure S1 Ascorbate intake and plasma levels of patients with metastatic melanoma receiving immunotherapy followed over time. A) Ascorbate intake and B) plasma ascorbate levels of the same patients in year 1 vs year 2 (n=16). C) Ascorbate intake and D) plasma ascorbate of the same patients measured over three subsequent appointments over 10 weeks (n=43).

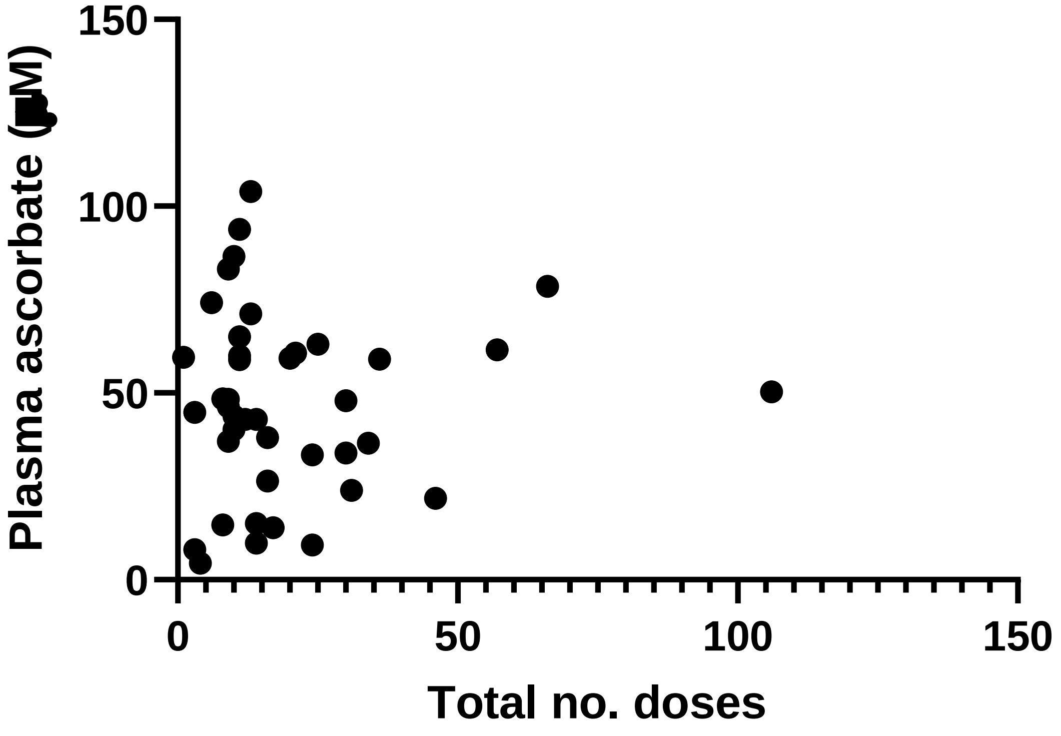

Figure S2 Relationship between the total number of immunotherapy doses received at the time of blood draw and plasma ascorbate concentration in patients with metastatic melanoma (n=41).
